# Clinical Presentation, Management and In-Hospital Outcome of Healthcare Personnel With COVID-19 Disease

**DOI:** 10.7759/cureus.10004

**Published:** 2020-08-24

**Authors:** Fazila-Tun-Nesa Malik, Mir Ishraquzzaman, Md Kalimuddin, Sohel Choudhury, Nazir Ahmed, Mohammad Badiuzzaman, Mir N Ahmed, Dhiman Banik, Tawfiq S Huq, Mohammad Abdullah Al Mamun

**Affiliations:** 1 Cardiology, National Heart Foundation Hospital & Research Institute, Dhaka, BGD; 2 Department of Epidemiology and Research, National Heart Foundation Hospital & Research Institute, Dhaka, BGD

**Keywords:** covid-19 disease, hcp, clinical presentation, in-hospital outcome

## Abstract

Objective

Healthcare personnel (HCP) are undoubtedly one of the major frontline fighters in the coronavirus disease 2019 (COVID-19) pandemic. Therefore, it comes as no surprise that many HCP have become infected by COVID-19 globally. The infection of HCP has received great attention in social media and is frequently reported from different parts of the world. However, there are few scientific reports addressing this aspect of the COVID-19 pandemic. The aim of this study was to evaluate the characteristics of clinical presentation, treatment, and outcome of COVID-19 infection among the HCP of our setting.

Methods

This cross-sectional study was performed in the National Heart Foundation Hospital & Research Institute of Bangladesh from April 29 to July 20, 2020. HCP employed in this hospital who experienced fever or respiratory symptoms or came in close contact with COVID-19 patients at home or their workplace were included in this study. The presence of COVID-19 disease was confirmed by real-time reverse transcriptase-polymerase chain reaction on nasopharyngeal samples. A total of 394 HCP were sampled and 139 had a positive corona test. Structured interviews were conducted to document symptoms for all HCP with confirmed COVID-19. Data analysis was performed in July 2020.

Results

Out of 1,409 HCP, 139 subjects tested positive for COVID-19. Among the HCP, infection rate was 9.86%. The mean age of the study population was 34.08±11.11 years (range: 20-69 yrs), of whom 82 (59%) were female. Most of this cohort were nurses (56 [40.3%]) and physicians (25 [18%]), and the remaining 58 (41.7%) were other staff. The mean duration of onset of symptoms to test was 2.89±2.07 days. The most common symptoms were fever (84.2%), fatigue (56.1%), cough (54%), body ache (39.6%), headache, and anosmia (38.8%). Most subjects had mild disease (125 [93%]), three (2.1%) of the HCP had moderate disease and one (0.7%) had severe disease. Ten of the HCP (7.2%) were asymptomatic. Most of them were treated either by ivermectin plus azithromycin or ivermectin plus doxycycline. Only 20 (14.4%) of the HCP were hospitalized, while others were treated either in home isolation (59.7%) or in institutional isolation (25.9%). Recovery was almost uneventful except one healthcare worker who died.

Conclusion

Most HCP had mild symptoms and a few of them were asymptomatic also. HCP with mild COVID-19 symptoms may be treated in home or institutional isolation. As they are a vulnerable group for infection, providing adequate protection to HCP is absolutely mandatory to safeguard them from this pandemic.

## Introduction

Severe acute respiratory syndrome coronavirus 2 (SARS-CoV-2) spreads rapidly by human-to-human transmission. The transmission is primarily by a combination of spread by droplet, direct and indirect contact and may possibly be airborne as well. Therefore, the highly contagious SARS-CoV-2 poses an unprecedented threat to frontline workers like health care personnel (HCP). HCP can easily be infected from patients while providing medical care to them. The absence of definite curative treatment or vaccines makes the scenario even more difficult. Protective measures against SARS-CoV-2 are of particular importance for HCP in direct contact with patients suffering from COVID-19 in the ambulatory as well as hospital setting. The European Society of Cardiology (ESC) and Society for Cardiovascular Angiography and Interventions (SCAI), American College of Cardiology (ACC), and the American College of Emergency Physicians (ACEP) guidance documents have suggested a high level of protection for HCP in the worst transmission scenario of SARS-CoV-2 infection [1,2]. The level of protection of HCP depends on patient risk status, setting and procedure performed. Newly admitted patients in a cardiology ward should be regarded as possibly infected by SARS-CoV-2 [1,2]. Incidence of HCP infection varies widely ranging from 1% - 29% [3-8]. This variation may be due to comparison of HCP with different populations, either with total number of HCP or with number of infected patients in the hospital. It needs to be remembered that HCP are infected either by nosocomial acquisition or community transmission.

The apparent higher rate of infection among HCP may be due to lack of awareness among staff, insufficient protective measures, social gatherings outside the workplace, or contact with known patients with COVID-19 in the community. The aim of this study was to evaluate demographic and clinical characteristics, management, and in-hospital outcome of COVID-19 among HCP with self-reported fever or respiratory symptoms in a tertiary cardiac care hospital.

## Materials and methods

Study design, setting, and population

The cross-sectional study was conducted in the non-COVID tertiary cardiac care hospital (National Heart Foundation Hospital & Research Institute, Dhaka, Bangladesh) employing 1,409 HCP. From April 29 to July 20, 2020, HCP with self-reported fever or respiratory symptoms or close contact with the patient at home or at the workplace in the last 10 days were tested for SARS-CoV-2 infection. Oral consent was obtained from all subjects. The study was approved by the Institutional Review Board (N.H.F.H.R.I/4-14/7/AD-1105).

Specimen collection process

Relevant hospital staff were trained for appropriate specimen collection (sufficiently deep swab), storage, packaging, and transport. Using a standardized technique, trained doctors or laboratory technicians obtained the nasopharyngeal swabs. After collection, swabs were placed in a transport medium and delivered to the laboratory. Diagnosis of COVID-19 was confirmed by real-time reverse transcription-polymerase chain reaction assay. Structured interviews were conducted to document symptoms for all HCP with confirmed COVID-19.

Statistical analysis

Sample size calculation and analysis for statistical significance were not performed because of the descriptive nature of this study. Categorical variables were presented as numbers and percentages and continuous data as mean and standard deviation. The analysis was performed with SPSS statistical software version 16.0 (SPSS Inc., Chicago, IL, USA). Data analysis was performed in July 2020.

## Results

By July 20, 2020, 139 of 1,409 HCP had tested positive for COVID-19 - an infection rate of 9.86%. The mean age of the study population was 34.08±11.11 years (range: 20-69 yrs), of whom 82 (59%) were female. Most of this cohort were nurses (56 [40.3%]) and physicians (25 [18%]), and the remaining 58 (41.7%) were other staff. Demographic characteristics of 139 HCP with confirmed COVID-19 are shown in Table 1.

**Table 1:** Demographic characteristics of COVID-19 infected HCP (n= 139) COVID-19: coronavirus disease 2019; HCP: healthcare personnel; SD: standard deviation

Characteristics	HCP, n (%)
Age	
Mean age ±SD	34.08 ±11.11 years
Sex	
Male	57 (41%)
Female	82 (59%)
Job category	
Physician	25 (18%)
Nurse	56 (40.3%)
Others HCP	58 (41.7%)

**Figure 1:**
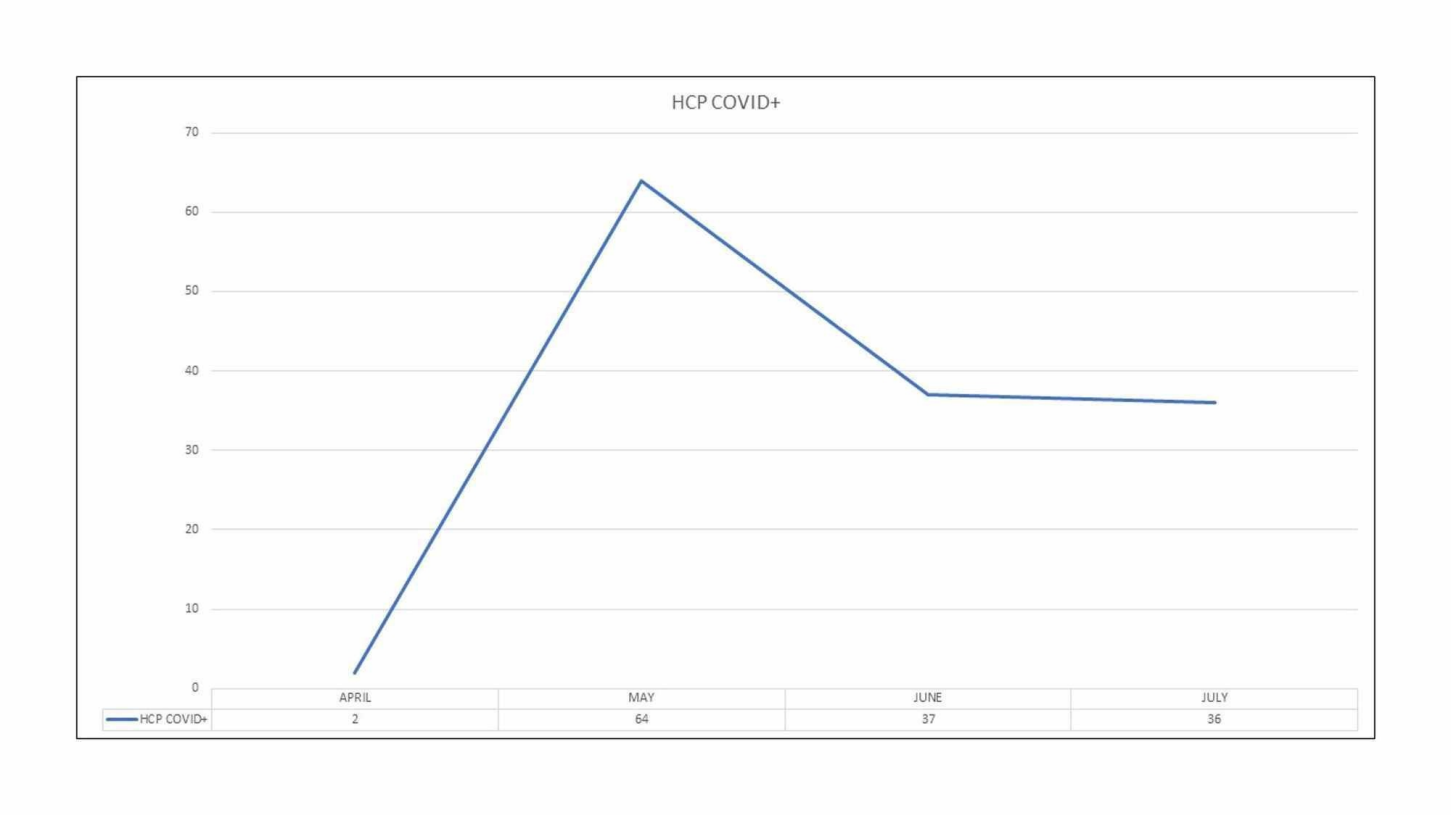
Month-wise distribution of COVID+ Health Care Personnel COVID+: Coronavirus Disease Positive; HCP: Health Care Personnel

It was also noted that the infection rate among HCP was highest in the month of May during the early stage of the pandemic in Bangladesh. Subsequently, the infection rate plateaued among the HCP in our hospital (Figure 1).

The common comorbidities among all HCP with infection were hypertension (15.1%), dyslipidemia (8.4%), diabetes (7.2%), chronic obstructive pulmonary disease, or bronchial asthma (7.2%) and cardiovascular disease (4.3%). Around 6.5% of HCP were smokers. Among the female staff, 8.5% were pregnant (7/82). Clinical characteristics of 139 HCP with confirmed COVID-19 are outlined in Table 2. The mean duration of onset of symptoms to test was 2.89±2.07 days. The most common symptoms were fever (84.2%), fatigue (56.1%), cough (54%), body ache (39.6%), headache, and anosmia (38.8%). Other symptoms were sore throat (36.7%), shortness of breath (26.6%), diarrhea (18.7%), and generalized itching (5.6%). Regarding disease severity, most of them had mild disease (93%), three (2.1%) had moderate disease and one (0.7%) had severe disease. Ten of the infected HCP (7.2%) were asymptomatic.

**Table 2:** Clinical characteristics of 139 HCP with confirmed COVID-19 HCP: healthcare personnel; COVID-19: coronavirus disease 2019; HTN: hypertension; DM: diabetes mellitus; COPD: chronic obstructive pulmonary disease; BA: Bronchial asthma

Characteristics	HCP, (n= 139)
Any comorbidity	
HTN	21 (15.1%)
DM	10 (7.2%)
COPD/BA	10 (7.2%)
Cardiovascular disease	6 (4.2%)
Dyslipidemia	12 8.4%)
Symptoms	
Fever	117 (84.2%)
Fatigue	78 (56.1%)
Cough	75 (54%)
Body ache	55 (39.6%)
Headache	54 (38.8%)
Anosmia	54 (38.8%)
Sore throat	51 (36.7%)
Shortness of breath	37 (26.6%)
Diarrhea	26 (18.7%)
Generalized itching	8 (5.6%)
Asymptomatic	10 (7.2%)
Disease severity	
Mild	125 (93%)
Moderate	3 (2.1%)
Severe	1 (0.7)
Mortality	1 (0.7%)

Around 5% (seven) of our study population took hydroxychloroquine as prophylaxis. Most HCP (105 [75.5%]) received ivermectin. Two of them (1.4%) received hydroxychloroquine and three (2.1%) received favipiravir. Regarding antibiotic treatment, 85 (61.2%) were treated by azithromycin and 46 (33.1%) by doxycycline. Thirteen (9.4%) HCP were given intravenous antibiotics (cephalosporin, meropenem or moxifloxacin alone or in combination). We used steroids in very few cases (six [4.2%]). Low molecular weight heparin was used in six (4.2%) HCP followed by newer oral anticoagulants. Most of them, therefore, received either ivermectin plus azithromycin or ivermectin plus doxycycline combination. We administered vitamin C and zinc to most of the HCP. Only 20 (14.4%) of the HCP were hospitalized, others were treated either in home isolation (59.7%) or in institutional isolation (25.9%). Recovery was almost uneventful except for one healthcare worker who died. He was a smoker and had triple vessel disease.

## Discussion

Our study was based in a large tertiary cardiac care hospital in Dhaka, Bangladesh. Many COVID-19-affected patients presented with cardiovascular symptoms. Also, many cardiac patients have underlying COVID-19 infection. Our hospital is a non-COVID hospital. However, during the pandemic, many patients were admitted who were subsequently diagnosed with COVID-19. Undoubtedly, taking care of these patients put the HCP of our hospital at risk infection with COVID-19.

A total of 394 HCP were sampled and 139 had a positive corona test. Among 1,409 HCP, 139 HCP tested positive for COVID-19 with an infection rate of 9.86%. The infection rate of HCP varies widely between hospitals, COVID and non-COVID hospitals, and different countries. The higher rate of infection among HCP of our hospital can be explained by several underlying factors. First, the study period was quite long - nearly three months as compared with other studies (e.g. three weeks [4], 40 days [3]). Second, community transmission also plays an important role. Some of the HCP were infected in the community during the initial stage of the pandemic due to lack of awareness, social gatherings outside the workplace, contact with known patients with COVID-19 in the community, and lack of personal protection outside the workplace. HCP took level II protection which includes disposable surgical cap, medical protection mask, work uniform, gown, disposable surgical gloves, and goggles or face shield for those who were in contact with patients. Third, the incubation time of COVID-19 ranges from 0-14 days. During the latent phase, it is difficult to recognize patients with the disease. Insufficient protective measures at the beginning of this pandemic put HCP at a higher risk. Fourth, in many patients the virulence of SARS-CoV-2 may not be severe. Many patients were asymptomatic or with few symptoms and rarely some patients presented with atypical symptoms [5]. Such patients could greatly jeopardize the health of staff in the hospital and in the community. Fifth, due to lack of disease knowledge, in the early period of this pandemic it was strenuous to identify patients with COVID-19.

A total of 7.2% (10) of HCP with COVID-19 were asymptomatic. In the early stage of the COVID era, asymptomatic patients could be one reason for the rapid spread of infection worldwide [9]. The transmission potential of asymptomatic carriers of SARS-CoV-2 was the same as the viral load detected in asymptomatic patients and was similar to that detected in symptomatic patients [10]. These asymptomatic HCP might become a risk factor for patients, colleagues, and the community. Therefore, early identification of asymptomatic carriers among HCP is of paramount importance and asymptomatic carriers should be isolated from family and colleagues to avoid cross-infection.

Lai et al. [3] assessed a group of HCP with predominantly nosocomial acquisition of SARS-CoV-2 in Wuhan, China where cases of COVID-19 were first reported. In that study, a total of 2,009 patients were diagnosed with COVID-19 in a tertiary hospital, 110 (5.5%) of whom were HCP, by February 9, 2020. Overall, 110 of 9,684 HCP tested positive for COVID-19, with an infection rate of 1.1%. Most of this cohort were nurses (62 [56.4%]) and physicians (26 [23.6%]), and the remaining 22 (20.0%) were healthcare assistants. Seventeen (15.5%) worked in fever clinics or wards, indicating an infection rate of 0.5% (17 of 3110) among frontline HCP. This relatively low infection rate is reassuring that personal protective equipment, if available, can protect frontline HCP directly caring for patients with COVID-19. A total of 93 of 6,574 non-frontline HCP (1.4%) were infected. Seventy-three (66.4%) worked in other clinical departments, and 20 (18.2%) worked in the hospital but did not interact with patients directly. These two groups had an infection rate of 1.65% (73 of 4433) and 0.99% (20 of 2012), [GP1] respectively. 

The prevalence and clinical manifestations of COVID-19 among HCP in two hospitals in the Netherlands were evaluated in the early phase of the pandemic [4]. In this study, HCP were also infected with SARS-CoV-2 in the community, in addition to nosocomial acquisition of SARS-CoV-2. Out of 9,705 HCP employed, 1,353 (14%) reported fever or respiratory symptoms and were tested in this cross-sectional study. Among them 86 HCP were infected with SARS-CoV-2 (6.36%), representing 1% of all HCP employed. Most patients were female (82.6%) and the median age was 49 years (range, 22-66 years). Among them 28% were nurses and 14% were physicians. Others with direct patient contact were 34% and without direct patient contact were 24%. Most HCP experienced mild disease.

Guan et al. [5] analyzed the data on 1,099 patients with laboratory-confirmed novel coronavirus (2019-nCoV) acute respiratory disease (ARD) from 552 hospitals in 31 provinces/provincial municipalities through January 29th, 2020 and revealed 2.09% were HCP.

Wu et al. [6] analyzed (updated through February 11, 2020) a total of 72,314 case records, among them 44,672 were classified as confirmed cases of COVID-19 (62%; diagnosis based on positive viral nucleic acid test result on throat swab samples). Among the 44,672 cases, a total of 1,716 were HCP (3.8%); 14.8% of cases were classified as severe or critical (247 of 1668) and there were five deaths.

On April 16, 2020, the Italian National Institute of Health (ISS) reported that 16,991 HCP had tested positive for SARS-CoV-2. These HCP had a median age of 48 years, and 68% were female and 32% were male. The infected HCP accounted for 10.7% of the total number of positive cases (n = 168,941) [7]. 

Wang et al. [8] evaluated 138 consecutive hospitalized patients with confirmed novel coronavirus (2019-nCoV) infected pneumonia (NCIP) at Zhongnan Hospital of Wuhan University in Wuhan, China, from January 1 to January 28, 2020. Of the 138 patients, 40 (29%) were HCP and 31 (77.5%) worked in general wards, seven (17.5%) in the emergency department, and two (5%) in the ICU.

Along with cardiac manifestations, one of the common extra-pulmonary manifestations of COVID-19 is diarrhea. Studies have shown that SARS-CoV-2 infects the gastro-intestinal (GI) tract via its viral receptor angiotensin converting enzyme 2 (ACE2), which is expressed on enterocytes of the ileum and colon [11]. A meta-analysis of 60 studies involving 4,243 COVID-19 patients from six countries revealed GI symptoms in 17.6% of the patients with anorexia (26.8%), diarrhea (12.5%), nausea/vomiting (10.2%), and abdominal pain/discomfort (9.2%) [12]. In the stool specimen of the first COVID-19 patient in the United States ribonucleic acid (RNA) of SARS-CoV-2 was detected, raising the concern for fecal-oral transmission of the virus [13]. In our study 18.7% (26) of patients had diarrhea. The incidence of diarrhea among HCP in other studies is 19%-35.5% [3,4].

Anosmia, a manifestation of olfactory dysfunction (OD), is a prominent sign of SARS-CoV-2 infection. Sudden onset of anosmia may be the only presenting symptom of patients with COVID-19 [14,15]. A mild symptom such as dry cough may be present before the onset of anosmia [16]. A retrospective study that included 114 confirmed COVID-19 patients and revealed 47% (54) of patients presented with anosmia. Anosmia generally developed 4.4 days after the onset of the infection and 98% of patients recovered within four weeks [17]. ACE2 is a functional receptor for SARS-CoV-2, and its expression and distribution in the nervous system suggest that SARS-CoV-2 can cause neurological manifestations through direct or indirect pathways [18]. Goblet cells and ciliated cells in the nasal mucosa may be the initial site of SARS-CoV-2 infection, indicating primary SARS-CoV-2 transmission is through infectious droplets [19]. Multiple evidence indicates that the nasal cavity is a vital area susceptible to SARS-CoV-2 infection [20]. Higher viral loads in the patient's nasal cavity than in the pharynx of both symptomatic and asymptomatic individuals hint at the nasal cavity as the first gateway for the initial infection [21]. Anosmia was present in about one-third (38.8%) of our study population.

In our study 93.7% of the infected HCP had mild disease. This is almost the same as other studies [3,4]. We also need to highlight that in our study most affected HCP were young adults (mean age only 34 years) as in other studies [3]. We postulate that the immunity of these young subjects was strong. Our HCP were identified with COVID-19 infection very early compared to non-HCP. Possibly the viral load could have been low in these subjects. However, we could not measure the viral load for our study population. The time between symptom onset and COVID test was shorter. The mean duration of onset of symptoms to test was 2.89±2.07 days. Treatment was initiated as early as possible. The above indicates that early diagnosis and treatment are essential for a better outcome of patients with COVID-19. Most of the HCP with infection were treated outside the hospital or in institutional isolation (85.6%). This might indicate that treating patients with mild symptoms outside a hospital setting can be a feasible method, particularly in the pandemic COVID era when there is a serious crisis of hospital beds.

On April 17 the latest estimate of medical doctor deaths reached 119 in Italy, which is 57.8% of total HCP deaths; followed by nurses at 16.5% (34), nurse aides at 8.3% (17), and dentists at 5.8% (12) [21]. Factors contributing to the elevated number of fatalities among HCP in Italy may be i) the sheer intensity of the COVID-19 outbreak; ii) the recruitment of elderly retired doctors and iii) shortages of personal protective equipment (PPE), particularly in non-hospital care [22]. Mortality per institute will definitely be low. As we mentioned previously, one HCP from our hospital died. The mortality rate among HCP was 0.7%, which is comparable with other studies [3] at 0.9%.

## Conclusions

Most of the HCP presented with mild symptoms. As they frequently have contact with COVID-19 patients and also have asymptomatic cases, periodic testing for COVID-19 among HCP is recommended to prevent further spread. HCP with mild COVID-19 symptoms may be treated in home or institutional isolation. As they are a vulnerable group for infection, providing adequate protection to HCP is absolutely mandatory to safeguard them from this pandemic. The infection of HCP may have a major negative impact on the capacity to treat patients, on the morale of professionals, and on public confidence during a pandemic, so protection of HCP is of paramount importance. Adequate protection is recommended to all HCP to fight against the highly contagious coronavirus.
